# Effects of Different Straw Return Modes on Soil Carbon, Nitrogen, and Greenhouse Gas Emissions in the Semiarid Maize Field

**DOI:** 10.3390/plants13172503

**Published:** 2024-09-06

**Authors:** Lu Hua, Zhenxing Yang, Wenqian Li, Yidong Zhao, Jie Xia, Wenyi Dong, Baoqing Chen

**Affiliations:** 1 College of Resources and Environment, Shanxi Agricultural University, Taiyuan 030031, China; hl20220312@163.com (L.H.); yang1981che@163.com (Z.Y.); 2 Institute of Environment and Sustainable Development in Agriculture, Chinese Academy of Agricultural Sciences, Beijing 100081, China; 82101235318@caas.cn; 3 Northern Agriculture and Livestock Husbandry Technology Innovation Center, Hohhot 010000, China; 4 Zibo Institute for Digital Agriculture and Rural Research, Zibo 255000, China; liwenqian0601h@163.com (W.L.); 15266726217@163.com (J.X.); 5 Zibo Academy of Agricultural Sciences, Zibo 255000, China

**Keywords:** deep burial straw return, dissolved organic carbon, nitrate nitrogen, maize grain, greenhouse gas emissions

## Abstract

Returning straw to the field is a crucial practice for enhancing soil quality and increasing efficient use of secondary crop products. However, maize straw has a higher carbon-to-nitrogen ratio compared to other crops. This can result in crop nitrogen loss when the straw is returned to the field. Therefore, it is crucial to explore how different methods of straw return affect maize (*Zea mays* L.) farmland. In this study, a field experiment was performed with three treatments (I, no straw returned, CK; II, direct straw return, SR; and III, straw returned in deep furrows, ISR) to explore the effects of the different straw return modes on soil carbon and nitrogen content and greenhouse gas emissions. The results indicated that the SR and ISR treatments increased the dissolved organic carbon (DOC) content in the topsoil (0–15 cm). Additionally, the ISR treatment boosted the contents of total nitrogen (TN), nitrate nitrogen (NO_3_^−^-N), ammonium nitrogen (NH_4_^+^-N), dissolved organic nitrogen (DON), and DOC in the subsurface soil (15–30 cm) compared with CK. When it comes to greenhouse gas emissions, the ISR treatment led to an increase in CO_2_ emissions. However, SR and ISR reduced N_2_O emissions, with ISR showing a more pronounced reduction. The ISR treatment significantly increased leaf and grain biomass compared to CK and SR. The correlation analyses showed that the yield was positively correlated with soil DOC, and soil greenhouse gas emission was correlated with soil NO_3_^−^-N. The ISR technology has great potential in sequestering soil organic matter, improving soil fertility, and realizing sustainable agricultural development.

## 1. Introduction

With advances in agricultural technology in China, crop production has risen significantly, leading to a dramatic increase in crop residues [1]. These residues are increasingly shifting from being used as bioenergy and animal feed to becoming agricultural waste. In semiarid regions with special climates and soil conditions, crop straw resources are often burned or underutilized. This not only results in the loss of valuable organic resources but also brings substantial pressure on the agroecosystems [2]. Therefore, how to effectively utilize straw resources has always been a major issue of wide social concern. Straw return to the field is a straightforward and highly effective method for managing secondary crop products. It also serves as a key strategy for maintaining soil organic matter, enhancing biological activity, improving physical properties, and increasing nutrient availability [3].

Maize straw often decomposes slowly and incompletely when returned to the field due to its high cellulose and hemicellulose content [4]. Therefore, optimizing straw return technology is crucial. Different straw return modes impact soil physicochemical properties and the straw decomposition process in various ways [5]. Straw mulching helps prevent soil erosion and retains soil carbon and nitrogen nutrients. However, straw mulch decomposes poorly and can increase the risk of pests and diseases [6,7]. Conventional composting can address the low decomposition rates more effectively, but handling large quantities of straw in the field can be challenging [8]. In contrast, directly burying straw in the soil significantly improves the decomposition efficiency and reduces the pest and disease risks [6]. However, shallow burial (0–20 cm) of straw can reduce soil pH around crop roots, which may hinder root growth. On the other hand, deep burial of straw can effectively enhance the root growth conditions, improve soil microbial communities, and boost soil fertility [9]. This approach is practical and reduces environmental impacts associated with traditional composting, aligning better with the needs of modern agricultural production.

Moreover, most crop residues have high carbon-to-nitrogen (C/N) ratios, which can lead to nitrogen competition between crops and soil microorganisms during decomposition processes [10]. If not effectively avoided, this competition will affect crop emergence and growth and deteriorate soil structure in the fields resulting in nutrient loss [11]. Straw with nitrogen fertilizer is crucial for regulating the soil C/N ratio and avoiding nitrogen competition [12]. It has been shown that the combination of straw and fertilizer application significantly enhanced the soil active organic carbon content and microbial function compared to straw application alone [13].

Straw return is an effective agricultural practice to promote soil organic matter, soil quality, and crop productivity [14]. It is also considered a protective measure that helps soil carbon sequestration and climate change mitigation [15]. Its impact on greenhouse gas (GHG) emissions has been widely discussed. Previous studies have generally shown that straw return increases CO_2_ emissions [16]. However, its effect on N_2_O emission has been inconsistent. Studies have shown that straw addition provides a sufficient carbon source for nitrate assimilation and denitrification and increases N_2_O emission [17]. Other studies have shown that returning straw to field can inhibit emission of N_2_O by stimulating bioimmobilization of mineral nitrogen or reducing N_2_O:N_2_ ratios during denitrification [18]. It is crucial to explore variations in GHG emissions under different straw return modes.

To summarize, while the effect of nitrogen application on the farmland environment under straw return to the field has been studied by previous researchers, there are few studies coupling these effects with composting. Therefore, this study utilized exogenous nitrogen with deep burial straw return, aiming to (1) investigate how a straw deep-burial mode influences soil carbon and nitrogen fractions and (2) compare the effects of different straw return modes on maize yields and GHG emissions. Based on the existing research foundation, we hypothesized that the deep burial of straw in the field would promote soil carbon and nitrogen accumulation, boost crop yields by accelerating the straw nutrients release, and at the same time mitigate the problem of soil GHG emissions. To verify this hypothesis, we conducted different straw return experiments to investigate the effects on soil carbon and nitrogen fractions, maize yield, and GHG emissions. By optimizing straw return technology for drylands, we can improve the soil quality and provide theoretical insights for the sustainable development of agriculture.

## 2. Materials and Methods

### 2.1. Experimental Site

**Figure 1:**
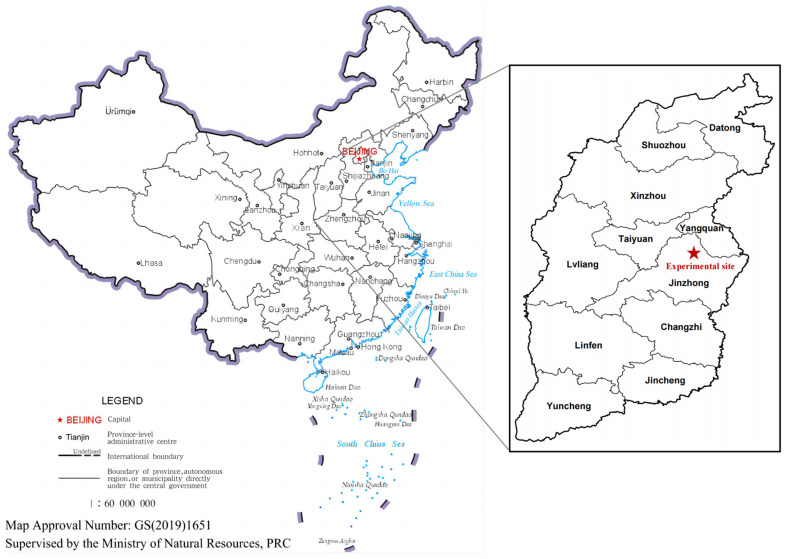
Location of the experimental site.

The field experiment was carried out at Shouyang Dryland Experimental Station, Shanxi Province (37°44′52″ N, 113°12′11″ E) (Figure 1), a dry farming area with an average annual precipitation of 481 mm and an average annual temperature of 8.4 °C in which the decomposition of straw was limited by dry and cold weather. Usually, maize is sown from late April to early May and harvested from late September to early October. After the harvest, there is a fallow period. The soil is classified as a Calcic Cambisol according to the FAO-UNESCO soil map [19], with a sandy loam texture, pH 7.8, soil bulk density of 1.38 g cm^−3^, field water capacity of 36.2%, organic carbon of 8.82 g kg^−1^ soil, and total nitrogen of 0.46 g kg^−1^ soil. In this study, the straw used was maize straw harvested in 2022. The organic carbon and total nitrogen contents of maize straw were 400 g kg^−1^ and 3.75 g kg^−1^, respectively.

### 2.2. Field Experimental Design

A field experiment was conducted during maize season (May 2023 to October 2023). There were three treatments: (1) no straw returned (CK), (2) direct straw return (SR), and (3) straw buried in deep furrows (ISR). Three replicated plots were set up for each treatment (6 × 6 m for each plot). Maize was sown at 60 cm row spacing and 30 cm plant spacing. Before sowing, rotary tillage was carried out at a depth of 15 to 20 cm. For the SR treatment, straw was chopped and evenly applied to the soils by plowing before maize cultivation (8 t ha^−1^). For the ISR treatment, straw was buried in the ditch by digging a 40 cm deep, 25 cm wide ditch in the middle of the two rows of maize. A slow-release blended fertilizer (total nutrients ≥ 45%, including 28% N, 12% P_2_O_5_, and 5% K_2_O) was applied at a rate of 804 kg ha^−1^ in all plots. In the ISR treatment, the fertilizer was applied to the deep ditch and the ground surface at a ratio of 1:1 (i.e., the application rate in the ditch was 402 kg ha^−1^, and the application rate on the ground surface was 402 kg ha^−1^), and the fertilizer was buried with the straw in the ditch. All plots were mulched with 10 μm thick polyethylene white mulch.

### 2.3. Sampling and Analysis Methods

#### 2.3.1. Soil Samples

Before the cultivation stage, the experimental plot was fallow. Soil samples were collected before planting (pre-plant), at the seedling stage (V1), jointing stage (V6), filling stage (R2), and maturity stage (R5). Soil samples (five replicates at the same depth per plot were mixed into one sample) were taken from 0–15 cm and 15–30 cm of each plot using a stainless-steel soil drill. Each layer of soil samples was mixed and brought back to the laboratory in sealed bags and stored at −20 °C. Some soil samples were used to determine soil NO_3_^−^-N, NH_4_^+^-N, DOC, and DON content, and some were air-dried and preserved for subsequent soil physicochemical analyses.

Soil NO_3_^−^-N and NH_4_^+^-N content were determined by KCl extraction and spectrophotometer colorimetry (UV-1900I Shimadzu Corporation, Tokyo, Japan) [20]. Soil TN content was determined by the Kjeldahl method (GK-600 Glkrui, Heze, Shandong, China) [21]. Soil DOC content was determined by water extraction and a TOC analyzer (TOC-VCPH Shimadzu Corporation, Tokyo, Japan) [22]. Soil DON content was obtained by calculating the difference between soil dissolved total nitrogen and soil inorganic nitrogen, and soil dissolved total nitrogen content was determined using the potassium persulfate oxidation and ultraviolet spectrophotometry method (UV-1900I Shimadzu Corporation, Tokyo, Japan) [23].

#### 2.3.2. Plant Samples

At the time of maize harvest, five maize plants were randomly selected in each plot and all above-ground parts were retrieved. Roots, leaves, stems, and grains of the retrieved maize plants were separated in the laboratory. Organs of the same species within the same plot were placed together. The samples were then placed in an oven and dried at 80 °C after killing at 105 °C until constant weight. Maize root biomass, stem biomass, leaf biomass, and grain biomass (yield) were weighed after drying the maize roots, stems, leaves, and grains.

#### 2.3.3. Determination of GHG Emissions

The static chamber method was used to collect GHG samples [24,25]. The chamber (φ0.2 × 0.26 m) was made of plastic. Gas samples were collected once every seven to ten days during the maize season. On every sampling day, two gas samples were collected from each chamber using 500 mL vacuum bags at 30-min intervals from 8:00 am to 11:00 am. At the same time, the static chamber temperature was recorded. The collected gas vials were taken back to the laboratory for analysis using a gas chromatograph. GHG emissions for unobserved dates between two adjacent measurements were calculated by multiplying the average emission flux of the two measurements by the time interval. The total emissions were then obtained by summing the observed values and the calculated values for the unobserved days on a day-by-day basis. The GHG emission flux calculation formula is shown in Equation (1):

(1) $$\begin{array}{c} F=\rho \times h\times dc/dt\times 273/(273+T) \end{array}$$

where *F* is the gas emission flux (μg m^−2^ h^−1^); *ρ* is the density of greenhouse gases at standard conditions; *h* is the height of the sampling box (m); *T* is the temperature inside the sampling box during sampling (°C); and *dc/dt* is the rate of change of the concentration of the gas measured in the sampling box (μL L^−1^ min^−1^).

The formula for calculating the total GHG emissions is shown in Equation (2):

(2) $$\begin{array}{c} Y=\sum_{i=1}^{n}(F_{i}+F_{i+1})/2\times \left(t_{i+1}-t_{i}\right)\times 24/100 \end{array}$$

where *Y* is the total amount of gas emitted from agricultural soils (kg ha^−1^); *F* is the gas emission flux (mg m^−2^ h^−1^); *i* is the ith time; $t_{i+1}-t_{i}$ is the number of days between sampling intervals; and *n* is the number of sampling times.

### 2.4. Data Analysis

The experimental data were entered and organized using Microsoft Office Excel 2016. A one-way ANOVA (one-way ANOVA) was performed on all data using IBM SPSS Statistics 27.0 (IBM Inc., Chicago, IL, USA) software. Duncan (α = 0.05) was used for multiple comparisons of the significance of differences between treatments for each indicator, and Origin2022 was used for graphing.

## 3. Results

### 3.1. Soil Dissolved Organic Carbon (DOC)

**Figure 2:**
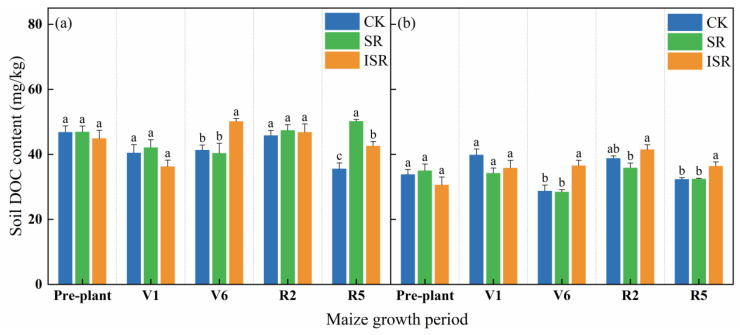
Soil DOC content of different straw return treatments. (**a**) The topsoil; (**b**) the subsurface soil. The data presented are the means of three replicates. Note: DOC, dissolved organic carbon; V1, seedling stage; V6, jointing stage; R2, filling stage; R5, maturity stage. Different lowercase letters over bars indicate significant differences at *p* < 0.05.

Figure 2 reflects the DOC content of the different layers of soils under the different straw return treatments at each maize fertility period. From the figure, it can be seen that with the growing maize, the variation trend of DOC content in the topsoil and the subsurface soil of each treatment was irregular. The topsoil DOC content of CK was the highest in the pre-plant period, and the topsoil DOC contents of the SR and ISR treatments were the highest in R5 and V6, respectively. The DOC content of the CK subsurface soil was the highest in the V1 period, and that of the SR and ISR treatments was the highest in the R2 period. In the V6 period, the topsoil DOC content in the ISR treatment was significantly increased by 21.3% and 24.2% compared with CK and SR. The subsurface soil DOC content in the ISR treatment increased by 27.1% and 28.6% compared with CK and SR. In the R2 period, the subsurface soil DOC content in the ISR treatment was 15.7% higher than that in the SR treatment. In the R5 period, the topsoil DOC content in the SR treatment was significantly increased by 41.1% and 17.8% compared with CK and ISR (*p* < 0.05). The subsurface soil DOC content in the ISR treatment increased by 12.5% and 12.1% compared with CK and SR, respectively.

### 3.2. Soil Nitrogen

#### 3.2.1. Soil Total Nitrogen (TN)

**Figure 3:**
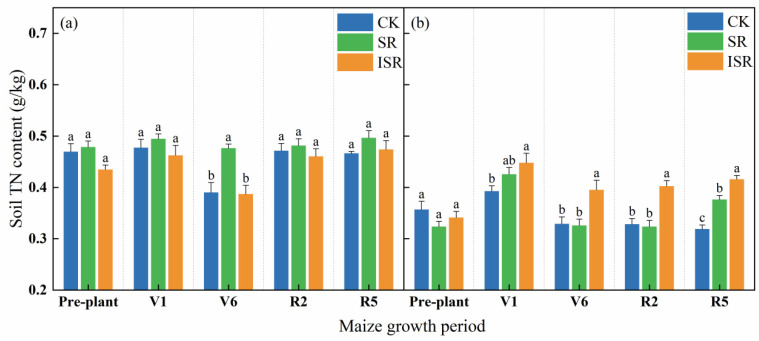
Soil TN content of different straw return treatments. (**a**) The topsoil; (**b**) the subsurface soil. Note: TN, total nitrogen; V1, seedling stage; V6, jointing stage; R2, filling stage; R5, maturity stage. Different lowercase letters over bars indicate significant differences at *p* < 0.05.

Figure 3 reflects the TN content of the two soil layers under the different straw return treatments at each maize fertility period. With the growth of maize, the topsoil TN content in the SR treatment varied little. The topsoil TN content in the CK and ISR treatments decreased during the V6 period. The subsurface soil TN content in the CK, SR, and ISR treatments exhibited an initial increase followed by a decrease. However, there was a resurgence in the total nitrogen content in the SR and ISR treatments during the R5 period. In the V1 period, the subsurface soil TN content in the ISR treatment was significantly increased by 14.0% compared with CK. In the V6 period, the topsoil TN content in the SR treatment increased by 22.1% and 23.1% compared with CK and ISR, respectively. The subsurface soil TN content in the ISR treatment increased by 20.2% and 21.3% compared with CK and SR, respectively. In the R2 period, the subsurface soil TN content in the ISR treatment was significantly higher by 22.6% and 24.3% compared to CK and SR. In the R5 period, the subsurface soil TN content in the ISR treatment was significantly increased by 30.4% and 10.5% compared with CK and SR, respectively. The subsurface soil TN content in the SR treatment was significantly increased by 18.0% compared with CK.

#### 3.2.2. Soil Nitrate Nitrogen (NO_3_^−^-N)

**Figure 4:**
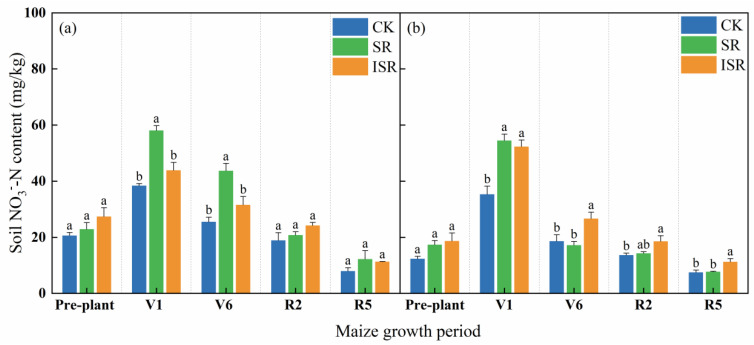
Soil NO_3_^−^-N content in different straw return treatments. (**a**) The topsoil; (**b**) the subsurface soil. Note: NO_3_^−^-N, nitrate nitrogen; V1, seedling stage; V6, jointing stage; R2, filling stage; R5, maturity stage. Different lowercase letters over bars indicate significant differences at *p* < 0.05.

Figure 4 reflects the NO_3_^−^-N content of the different layers of soils under the different straw return treatments at each maize fertility period. As can be seen from the figure, soil NO_3_^−^-N of all soil layers under the different treatments showed a changing trend of first increasing and then decreasing. In the V1 period, the topsoil NO_3_^−^-N content in the SR treatment increased by 51.3% and 32.4% compared with CK and ISR, respectively. The subsurface soil NO_3_^−^-N contents in the SR and ISR treatments were significantly increased by 54.4% and 48.3% compared with CK. In the V6 period, the topsoil NO_3_^−^-N content in the SR treatment increased by 71.4% and 38.6% compared with CK and ISR, respectively. The subsurface soil NO_3_^−^-N content in the ISR treatment increased by 43.0% and 55.1% compared with CK and SR, respectively. In the R2 period, subsurface soil NO_3_^−^-N in the ISR treatment was 35.9% higher than that in CK. In the R5 period, the subsurface soil NO_3_^−^-N content in the ISR treatment was significantly increased by 50.3% compared with CK.

#### 3.2.3. Soil Ammonium Nitrogen (NH_4_^+^-N)

**Figure 5:**
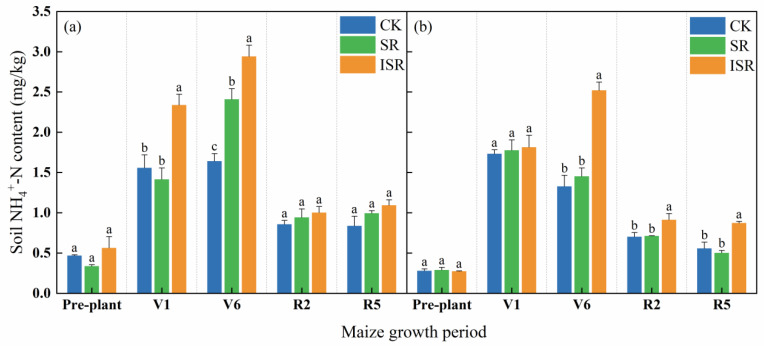
Soil NH_4_^+^-N content in different straw return treatments. (**a**) The topsoil; (**b**) the subsurface soil. Note: NH_4_^+^-N, ammonium nitrogen; V1, seedling stage; V6, jointing stage; R2, filling stage; R5, maturity stage. Different lowercase letters over bars indicate significant differences at *p* < 0.05.

Figure 5 reflects that soil NH_4_^+^-N of all treatments showed a trend of first increasing and then decreasing. In the V1 period, the topsoil NH_4_^+^-N content in the ISR treatment increased by 50.1% and 65.3% compared with CK and SR, respectively. In the V6 period, the NH_4_^+^-N content of the topsoil and the subsurface soil in the ISR treatment was significantly higher than that of SR and CK, and the topsoil NH_4_^+^-N content in the SR treatment was significantly higher than that of CK. In the R2 period, the subsurface soil NH_4_^+^-N content in the ISR treatment increased by 29.7% and 28.1% compared with CK and SR, respectively. In the R5 period, the subsurface soil NH_4_^+^-N content in the ISR treatment increased by 56.8% and 74.2% compared with CK and SR, respectively.

#### 3.2.4. Soil Dissolved Organic Nitrogen (DON)

**Figure 6:**
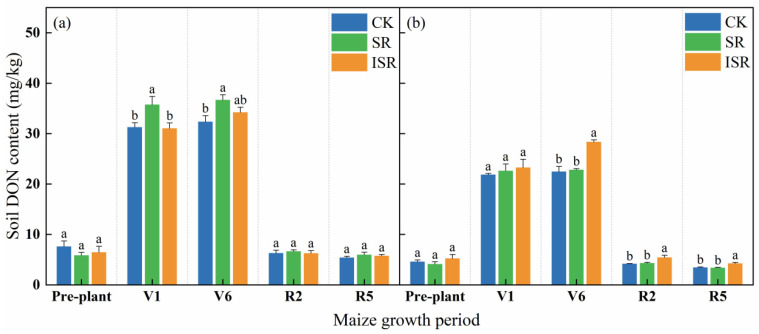
Soil DON content in different straw return treatments. (**a**) The topsoil; (**b**) the subsurface soil. Note: DON, dissolved organic nitrogen; V1, seedling stage; V6, jointing stage; R2, filling stage; R5, maturity stage. Different lowercase letters over bars indicate significant differences at *p* < 0.05.

Figure 6 reflects that soil DON of all treatments showed a trend of first increasing and then decreasing. In the V1 period, the topsoil DON content in the SR treatment increased by 14.3% and 15.1% compared with CK and ISR, respectively. In the V6 period, the topsoil DON content in the SR treatment was significantly increased by 13.3% compared with CK. The subsurface soil DON content in the ISR treatment increased by 26.3% and 24.3% compared with CK and SR, respectively. In the R5 period, the subsurface soil DON content in the ISR treatment increased by 22.5% and 25.2% compared with CK and SR, respectively.

### 3.3. Biomass of Different Parts of Maize

**Figure 7:**
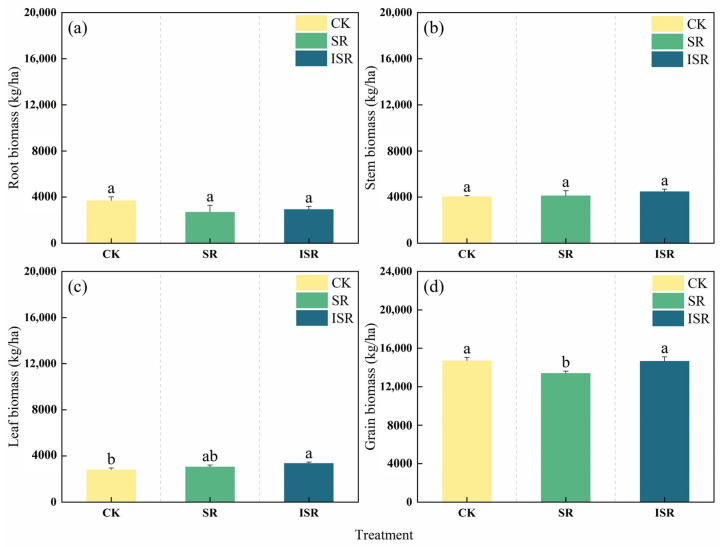
Biomasses of maize organs in different straw return modes. (**a**) Root biomass; (**b**) stem biomass; (**c**) leaf biomass; and (**d**) grain biomass. Note: CK, no straw returned; SR, direct straw return; ISR, straw returned in deep furrows. Different lowercase letters over bars indicate significant differences at *p* < 0.05.

Figure 7 reflects the biomasses of each organ for the different treatments. From the figure, it can be seen that the maize grain biomass of the CK, SR, and ISR treatments were 14.71 t ha^−1^, 13.41 t ha^−1^, and 14.67 t ha^−1^, respectively. Among them, the yields of the CK and ISR treatments were significantly increased by 9.7% and 9.4% (*p* < 0.05) as compared to the SR treatment, but the difference in the yield increase between them was not significant. In addition to the grain biomass, the root, stem, and leaf biomasses of different treatments ranged from 2713.3–3705.1 kg ha^−1^, 4060.0–4490.7 kg ha^−1^, and 2817.5–3364.4 kg ha^−1^, respectively. The leaf biomass was significantly increased by 19.4% in the ISR treatment as compared to CK. There was no significant difference in the root and stem biomasses among all treatments. The total biomass of the CK, SR, and ISR treatments was 25,291.6 kg ha^−1^, 23,312.6 kg ha^−1^, and 25,473.7 kg ha^−1^, respectively. Significantly higher maize biomass was observed in the CK and ISR treatments by 8.5% and 9.3%, respectively, compared to SR (*p* < 0.05).

### 3.4. Soil GHG Emissions

**Figure 8:**
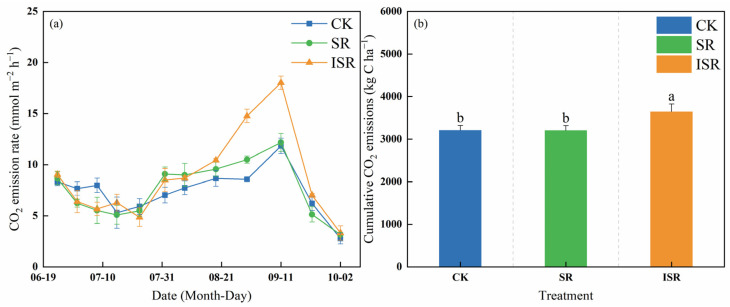
Dynamics of CO_2_ emission rate and cumulative emissions from soils. (**a**) CO_2_ emission flux; (**b**) cumulative CO_2_ emissions. Note: CK, no straw returned; SR, direct straw return; ISR, straw returned in deep furrows. Different lowercase letters over bars indicate significant differences at *p* < 0.05.

Figure 8 reflects the carbon dioxide (CO_2_) emission rate and the cumulative soil CO_2_ emissions under the different straw return modes. The trend of the soil CO_2_ emission rate was the same during the growth of maize in the different treatments, which all showed the trend of increasing then decreasing. The highest fluxes peaked in the different treatments with a range of 11.84–18.02 mmol m^−2^ h^−1^ during the later stages of growth. The soil cumulative CO_2_ emissions of the different treatments ranged from 3212.1 kg C ha^−1^ to 3645.6 kg C ha^−1^, with the cumulative CO_2_ emissions of the ISR treatment significantly (*p* < 0.05) increased by 13.5% and 13.6% compared with the the CK and SR treatments, respectively.

**Figure 9:**
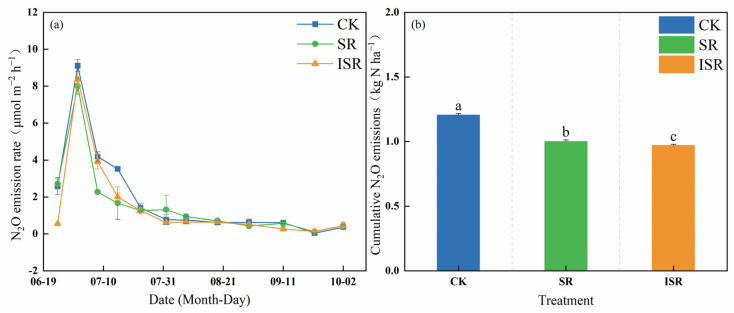
Dynamics of soil N_2_O emission rates and cumulative emissions. (**a**) N_2_O emission flux; (**b**) cumulative N_2_O emissions. Note: CK, no straw returned; SR, direct straw return; ISR, straw returned in deep furrows. Different lowercase letters over bars indicate significant differences at *p* < 0.05.

Figure 9 represents the nitrous oxide (N_2_O) emission rate and the soil N_2_O cumulative emissions under the different straw return modes. The soil N_2_O emission rate showed a general trend of prolonged decrease during the growth of maize in the different treatments, and its peak appeared on July 1st. The soil cumulative N_2_O emissions of the different treatments ranged from 0.97 kg N ha^−1^ to 1.21 kg N ha^−1^, with the SR and ISR treatments significantly reducing N_2_O emissions by 16.9% and 19.4%, respectively, compared with the CK treatment. In addition, the ISR treatment significantly reduced N_2_O emission by 3.0% (*p* < 0.05) compared to the SR treatment.

### 3.5. Correlations between GHG Emissions, Maize Biomass, and Soil Chemical Parameters

**Figure 10:**
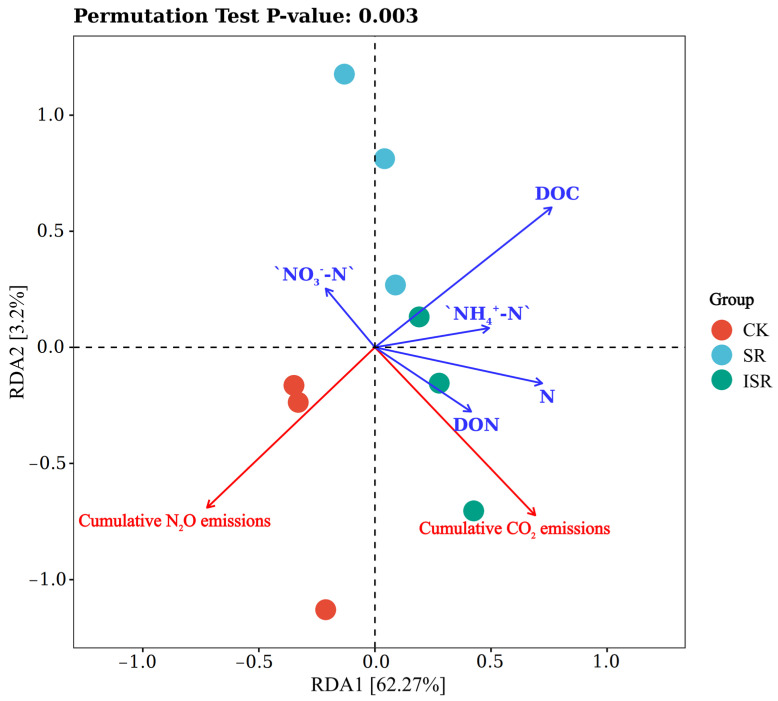
RDA analysis between different treatments of soil physicochemical properties and maize yield and biomass. Note: DOC, dissolved organic carbon; DON, dissolved organic nitrogen; NO_3_^−^-N, nitrate nitrogen; NH_4_^+^-N, ammonium nitrogen.

The relationships between the soil GHG emissions and changes in the soil chemical parameters (TN, NO_3_^−^-N, NH_4_^+^-N, DON, and DOC) were analyzed using redundancy (RDA) analysis (Figure 10). The first two coordinate axes of the RDA could explain 65.47% of the total variance. The two axes better reflected the correlation that existed between the GHG emissions and the soil chemical parameters. The results of the RDA likewise indicated a significant effect of the soil chemical parameters on the soil GHG emissions (*p* = 0.008). Through the angular relationships among the factors, it was observed that soil CO_2_ emission was significantly correlated with soil DON, TN, and NO_3_^−^-N. Soil N_2_O emission was significantly correlated with soil DOC and NH_4_^+^-N. These indicate that the sensitivity of different greenhouse gases (CO_2_ and N_2_O) to the soil chemical parameters varied.

**Figure 11:**
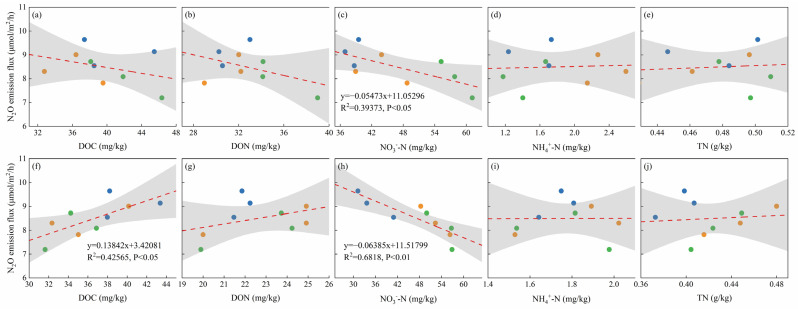
Correlations between soil N_2_O emission flux and soil factors. (**a**) Topsoil DOC; (**b**) topsoil DON; (**c**) topsoil NO_3_^−^-N; (**d**) topsoil NH_4_^+^-N; (**e**) topsoil TN; (**f**) subsurface soil DOC; (**g**) subsurface soil DON; (**h**) subsurface soil NO_3_^−^-N; (**i**) subsurface soil NH_4_^+^-N; and (**j**) subsurface soil TN. Note: DOC, dissolved organic carbon; DON, dissolved organic nitrogen; NO_3_^−^-N, nitrate nitrogen; NH_4_^+^-N, ammonium nitrogen; TN, total nitrogen.

**Figure 12:**
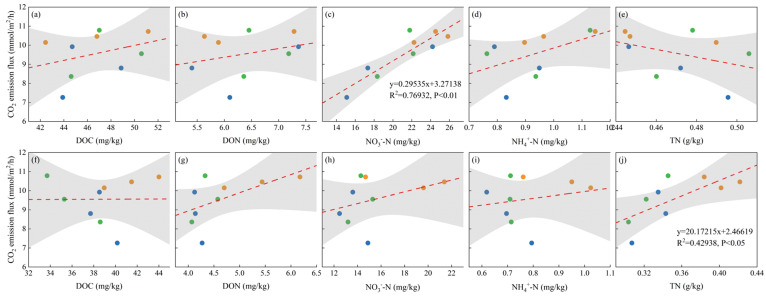
Correlations between soil CO_2_ emission flux and soil factors. (**a**) Topsoil DOC; (**b**) topsoil DON; (**c**) topsoil NO_3_^−^-N; (**d**) topsoil NH_4_^+^-N; (**e**) topsoil TN; (**f**) subsurface soil DOC; (**g**) subsurface soil DON; (**h**) subsurface soil NO_3_^−^-N; (**i**) subsurface soil NH_4_^+^-N; and (**j**) subsurface soil TN. Note: DOC, dissolved organic carbon; DON, dissolved organic nitrogen; NO_3_^−^-N, nitrate nitrogen; NH_4_^+^-N, ammonium nitrogen; TN, total nitrogen.

As shown in Figure 11 and Figure 12, the relationships between the soil GHG emissions (CO_2_ and N_2_O) and the soil chemical parameters (DOC, DON, NO_3_^−^-N, NH_4_^+^-N, and TN) were linearly fitted. The results showed that in the topsoil, the N_2_O emission flux was negatively correlated with soil NO_3_^−^-N, and the N_2_O emission rate decreased linearly with increases in the soil NO_3_^−^-N concentration (R^2^ = 0.39, *p* < 0.05). The soil CO_2_ emission flux was positively correlated with soil NO_3_^−^-N, and the CO_2_ emission rate increased linearly with increases in the soil NO_3_^−^-N concentration (R^2^ = 0.77, *p* < 0.01). In the subsurface soil, the soil N_2_O emission flux was positively correlated with soil DOC and negatively correlated with NO_3_^−^-N. The N_2_O emission rate increased with increases in the soil DOC concentration (R^2^ = 0.43, *p* < 0.05) and decreased linearly with increases in the NO_3_^−^-N concentration (R^2^ = 0.68, *p* < 0.01). The soil CO_2_ emission flux was positively correlated with soil TN, and the CO_2_ emission rate increased linearly with increases in the soil TN concentration (R^2^ = 0.43, *p* < 0.05).

**Figure 13:**
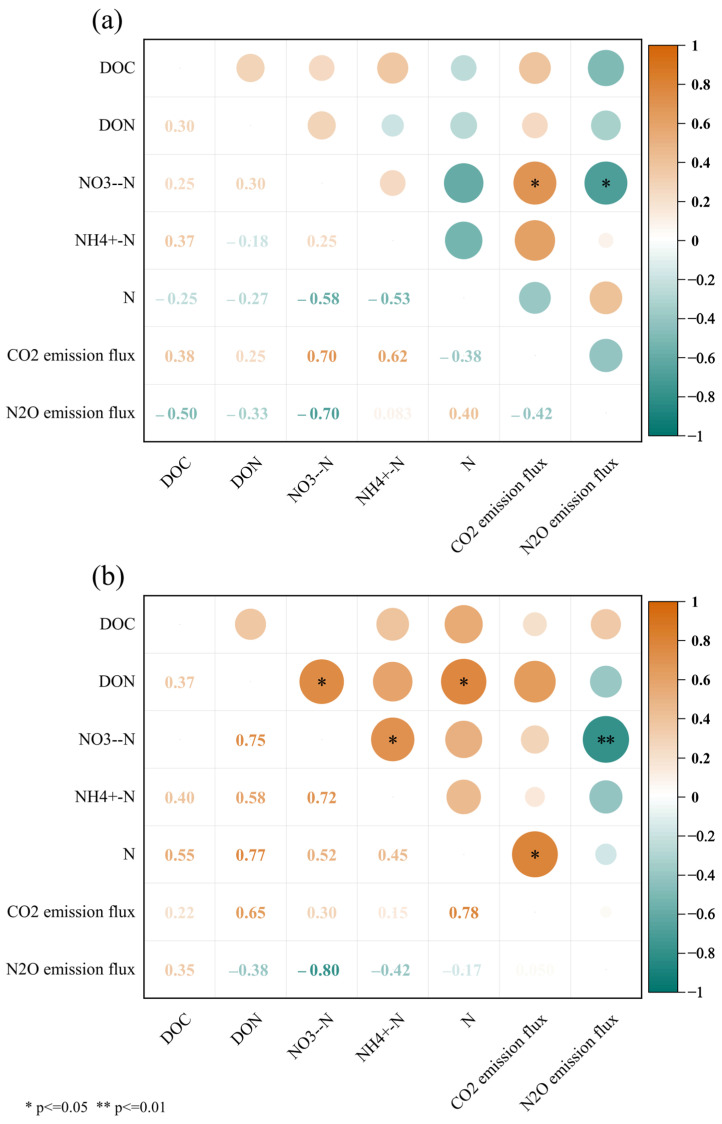
Correlations between soil chemical parameters and GHG (CO_2_ and N_2_O) emission fluxes under different treatments. (**a**) Topsoil, (**b**) subsurface soil. Note: DOC, dissolved organic carbon; DON, dissolved organic nitrogen; NO_3_^−^-N, nitrate nitrogen; NH_4_^+^-N, ammonium nitrogen; N, total nitrogen.

The analysis used the Spearman correlation heatmap to examine the relationships between the soil chemical parameters and the GHG emission fluxes (Figure 13). The results indicated a strong positive correlation between the soil CO_2_ emission flux and the NO_3_^−^-N content in the topsoil (+0.70). Conversely, a significant negative correlation was found between the N_2_O emission flux and the NO_3_^−^-N content in the topsoil (−0.70). In the subsurface soil, a significant positive correlation was observed between the soil CO_2_ emission flux and soil TN (+0.78). Additionally, a significant negative correlation was found between the N_2_O emission flux and the NO_3_^−^-N content (−0.80).

**Figure 14:**
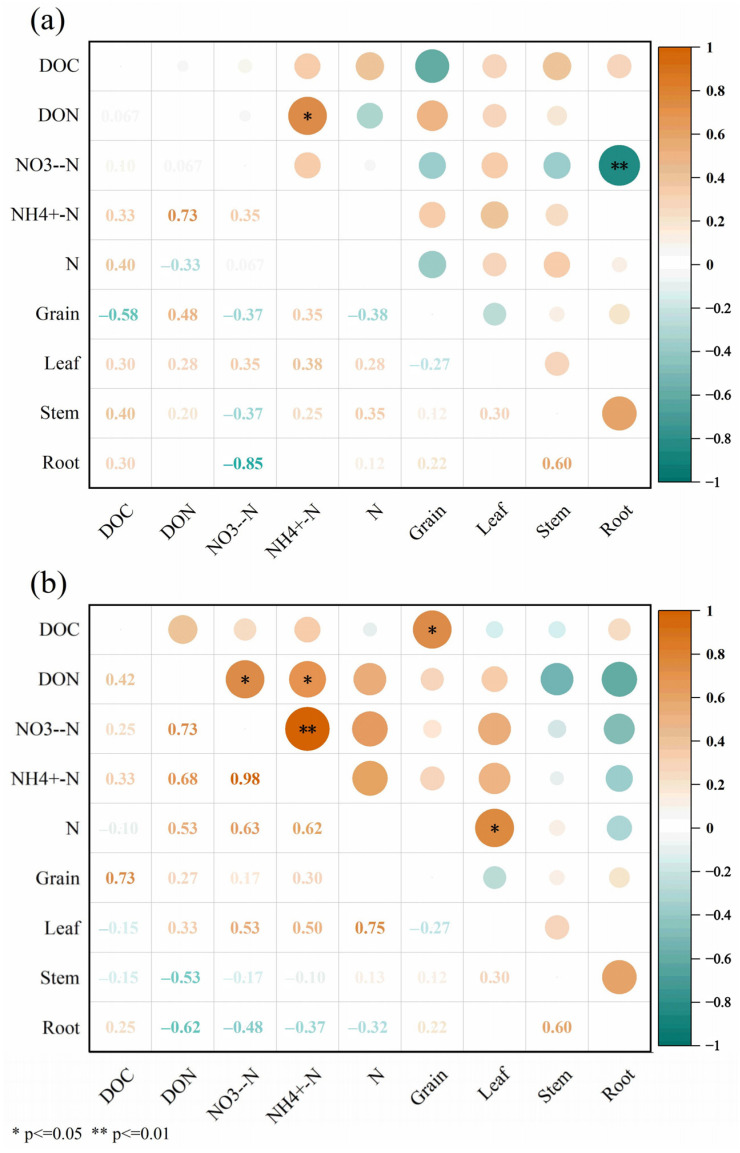
Correlations between soil chemical parameters and maize biomass under different treatments. (**a**) Topsoil, (**b**) subsurface soil. Note: DOC, dissolved organic carbon; DON, dissolved organic nitrogen; NO_3_^−^-N, nitrate nitrogen; NH_4_^+^-N, ammonium nitrogen; N, total nitrogen.

The Spearman correlation heatmap analysis was used to investigate the relationships between the soil chemical parameters and the maize biomasses (Figure 14). The results showed that soil DON was significantly positively correlated with the NH_4_^+^-N content in the topsoil (+0.73). The soil NO_3_^−^-N content was significantly negatively correlated with the root biomass (−0.70). In the subsurface soil, there were positive correlations among soil DON, NO_3_^−^-N, and NH_4_^+^-N. The correlation coefficients were all between 0.73–0.98. In addition, the results also showed that the grain biomass was positively correlated with the soil DOC content (+0.73). The leaf biomass was positively correlated with the soil TN content (+0.75).

## 4. Discussion

### 4.1. Effects of Different Straw Return Modes on Soil Dissolved Organic Carbon

Soil carbon is an important index to characterize changes in soil fertility, which directly or indirectly affects the physical, chemical, and biological properties of soil. Straw return modes are processes that convert organic carbon from crop carbon pools into soil carbon pools, affecting soil organic carbon content throughout soil horizons [26]. However, short- and medium-term changes in soil carbon pools are difficult to distinguish due to spatial and temporal differences in the persistence of soil organic carbon. DOC is one of the important components of soil unstable organic carbon, which is highly influenced by soil chemical parameters and responds rapidly to land management measures. Thus it is considered one of the sensitive indicators reflecting changes in soil organic carbon [27]. Our study showed that the SR and ISR treatments increased topsoil DOC compared with CK; ISR also increased subsurface soil DOC. This was attributed to the fact that soil microorganisms can decompose straw to release a large amount of dissolved organic matter (e.g., polysaccharides, peptides, and aliphatic compounds), which promotes the accumulation of DOC [28]. Moreover, the ISR treatment not only provided sufficient nitrogen for soil microorganisms, but also promoted the accumulation of active organic carbon in the soil as a result of the formation of composting zones between the crop rows [29].

### 4.2. Effects of Different Straw Return Modes on Soil Nitrogen

Nitrogen is a critical soil component that limits terrestrial primary productivity and is essential for crop growth, significantly influencing crop development and yield [30]. Straw return modes directly increase carbon and nitrogen sources for the soil, provide essential nitrogen for soil microbial activities, and promote the activity of soil microorganisms to decompose straws, thus releasing more nitrogen nutrients [31]. In our study, both the SR and ISR treatments elevated the soil TN content in the subsurface soil, which was in agreement with the previous study [6]. Among them, the ISR treatment had a more significant effect on enhancing the soil nitrogen nutrients, likely because the exogenous fertilization directly stimulated microbial straw decomposition processes in the subsurface soil. The SR and ISR treatments in all soil layers had different rebound effects after the filling stage. This variation was likely due to the reduced nitrogen demand in the late stage of maize growth and the release of straw nitrogen causing the accumulation of soil nitrogen [32].

Plant uptake and utilization of nitrogen is mainly in the form of inorganic nitrogen (NH_4_^+^-N and NO_3_^−^-N) [33]. The level of inorganic nitrogen is an important indicator for evaluating the availability of soil nitrogen. Straw return promoted the soil NH_4_^+^-N content in our study. Xu et al. [34] demonstrated that in agroecosystems, a portion of fertilizer nitrogen and straw nitrogen not taken up by crops is mainly converted to NH_4_^+^-N, which corroborates our findings. Moreover, Chen et al. [35] demonstrated that soil with straw return had higher nitrogen mineralization potential and mineral nitrogen content, indicating that long-term straw return significantly enhanced soil nitrogen supply capacity. However, the increase of NH_4_^+^-N content may lead to an increase in ammonium loss [36]. Straw addition caused rapid increases in soil NH_4_^+^-N and NO_3_^−^-N in the SR and ISR treatments during the pre-plant period of maize. The soil NO_3_^−^-N content in the V1 period was the highest. This may be because part of the nitrogen may have been released from organic bonds. So maize not only had enough nitrogen but also had a lot of excess. Especially in the ISR treatment, it was possible that greater mineralization of the original organic matter could play an important role. With the growth of maize, soil mineral nitrogen began to decline due to elevated crop nitrogen requirements [37]. We observed that in the subsurface soil, the SR and ISR treatments elevated NO_3_^−^-N compared with CK because the straw decomposition provided an additional nitrogen source. Furthermore, the ISR treatment provided an additional nitrogen source while forming a decomposition zone in the subsurface soil to accelerate the straw decomposition, which increased the soil NO_3_^−^-N accumulation even more [29].

Soil DON is one of the main components of soil dissolved organic matter, which is an intermediate product of soil microbial life activities and organic matter transformation. Soil DON content is a comprehensive reflection of soil microbial decomposition and utilization. In our study, the content of soil DON in each soil layer of each treatment increased first and then decreased. The significant increase in the DON content may be attributed to the enhancement of carbon and energy for microorganisms during the decomposition of the straw. This process was beneficial for converting straw carbon into soil active organic carbon and inorganic nitrogen into DON [38]. In addition, the straw return combined with the appropriate nitrogen fertilizer promoted activation and decomposition of insoluble substances in the soil and increased soil dissolved substances such as DOC and DON [39]. At the later stage of maize growth, nitrogen uptake by roots increased rapidly with the root biomass [40]. The soil DON content decreased with the slowing of the straw decomposition and the utilization of available nitrogen by the maize.

### 4.3. Effect of Different Straw Return Modes on Yield

Straw returned to the fields affects crop yield in multiple ways by improving soil structure and physicochemical properties, promoting microbial activity, and enhancing soil fertility [41]. Moreover, nitrogen absorption, nitrogen use efficiency, and maize yield could be improved significantly by applying fertilizer with straw [42]. However, our study found that SR resulted in a lower yield compared to CK, which could be analyzed as a result of soil moisture limitation. The experimental site belongs to a semiarid region with low annual precipitation. SR reduces the water holding capacity of the topsoil, while straw decomposition also absorbs soil moisture. This results in a reduction in the water supplied to the crop for growth [8], which leads to a reduction in the maize yield. In addition, soil tillage management can affect soil structure, which can cause larger changes in crop productivity. In our study, we found that the maize yield was significantly higher in the ISR treatment than in SR, which may have been due to the ability of deep tillage to break up poorly aerated and permeable subsurface soil compared to rotary tillage, which helped maize rooting down and contributed to maize yield growth [43]. This study was conducted for only one year, and the long-term effects of ISR on maize yield need to be further investigated.

### 4.4. Effect of Different Straw Return Modes on GHG Emissions

The addition of maize straw to a field provides rich carbon and nitrogen energy sources for microorganisms and increases the activity of microorganisms, which in turn accelerates soil microbial respiration to release CO_2_ [14]. Liu et al. [44] showed that the soil porosity produced by straw returning to the field is favorable for the emission of CO_2_, and the emission of soil CO_2_ increases. Our results were consistent with the previous authors, that ISR increased the cumulative CO_2_ emissions. The reason is that the application of nitrogen sources directly relieves nitrogen tension while accelerating straw decomposition and accelerating soil respiration thus releasing more CO_2_ [45]. We found that the different straw-return treatments showed peak CO_2_ emissions at the late stage of maize growth, which may have been related to frequent rainfall in the late period. Precipitation increases soil moisture and provides sufficient water for soil microbial activities. This further accelerates the rate of microbial respiration and organic carbon decomposition, increasing soil CO_2_ emissions [25]. In addition, Wu et al. [46] showed that soil CO_2_ emissions had a greater response to increased rainfall under drought conditions.

In addition, a large number of studies have shown that straw return to the field affects the nitrogen cycling process in the soil by influencing soil gas permeability and soil microbial activity, thereby increasing N_2_O emissions [45,47]. However, our study found that the straw addition significantly reduced the soil N_2_O emission during crop growth in agreement with Li et al. [48]. The reason may be that the straw addition accelerated the microbial carbon and nitrogen turnover in the soil, creating reducing conditions that facilitated the reduction of N_2_O to N_2_ [14]. Furthermore, the emission reduction effect of the ISR treatment was more significant. This may be because, under the condition of the deep straw burial, N_2_O diffusion must cross the thicker soil layer. This increased soil residence time favors the conversion of N_2_O into N_2_, which is conducive to the reduction in N_2_O emissions [49]. N_2_O is an important GHG in the atmosphere with a warming potential 273 times higher than that of CO_2_ on a century timescale [50]. Therefore, ISR had a certain effect of slowing down the greenhouse effect in our study. At the early stage of maize, there was an emission peak of N_2_O in all treatments, which was attributed to the fact that the nitrogen fertilizer application provided energy to microorganisms, which promoted the activity of soil nitrifying and denitrifying bacteria thus increasing N_2_O emission [51]. In the middle and late periods of maize growth, the easily decomposed substances in the straw were gradually exhausted, and the N_2_O emission rates of the different treatments gradually converged.

### 4.5. Correlation Analysis of Soil Carbon and Nitrogen Properties with GHG Emissions and Maize Yield

Most studies have shown that soil organic carbon is a key factor in influencing crop yield and improving soil quality [52]. However, DOC is the most mobile and available organic carbon component compared to organic carbon, which is more sensitive to agricultural practices such as fertilization [12]. Soil DOC has a great impact on regulating soil nutrients and is highly correlated with intrinsic soil productivity. Our study found that soil DOC was the main soil factor affecting the maize yield in the agricultural fields. While providing a labile carbon source, soil DOC accumulation can further promote crop growth and development by enhancing soil aggregate stability and improving soil physical properties [53].

NO_3_^−^-N is one of the nitrogen sources that can be directly absorbed and utilized by plants and plays an important role in both soil nitrogen cycling and influencing plant growth. High NO_3_^−^-N accumulation reduces nitrogen utilization efficiency and increases the risk of NO_3_^−^-N leaching [54], thus limiting nutrient uptake by crop roots and affecting root growth [55]. This situation was corroborated in our study, where the maize root biomass was negatively correlated with NO_3_^−^-N in the topsoil. At the same time, the soil denitrification rate increased with increasing root biomass during crop growth, which caused a decrease in the NO_3_^−^-N content [56]. Moreover, a positive correlation was observed between NH_4_^+^-N and DON in the topsoil, and a positive correlation was observed between NO_3_^−^-N, NH_4_^+^-N, and DON in the subsurface soil, which reflected the dynamic balance of the soil nitrogen cycle.

In farmland ecosystems, plant growth mainly depends on nitrogen supplementation, with nitrogen enrichment promoting plant growth and soil respiration [57]. We revealed a positive correlation between the CO_2_ emissions, soil NO_3_^−^-N, and the TN contents, consistent with the findings of Dai X et al. [58]. The increase in the soil nitrogen content promoted sufficient nutrient uptake by the maize roots. In turn, this enhanced root exudate production and increased soil respiration, leading to the emission of CO_2_ [59]. Generally, soil labile organic carbon serves as an energy source for soil microorganisms, thereby enhancing microbial activity. Straw provides easily accessible carbon and nitrogen sources to the soil, which can subsequently influence soil GHG emissions [44]. Our study observed that the N_2_O emissions were positively correlated with DOC, which is because DOC is the most readily available carbon source for microorganisms. The increase in the DOC content accelerated the soil microbial nitrification and denitrification processes, leading to higher N_2_O emissions [60]. Conversely, the N_2_O emissions were negatively correlated with NO_3_^−^-N. This may be because high NO_3_^−^-N promotes denitrification in the soil and accelerates the conversion of more N_2_O to N_2_, thereby reducing N_2_O emissions. Additionally, NO_3_^−^-N is the substrate of denitrification [46], and high N_2_O emission can directly lead to reduction of soil NO_3_^−^-N. Zhou et al. [61] reported that applying cellulose as a carbon source into soil can increase soil NO_3_^−^-N while suppressing soil N_2_O emission.

## 5. Conclusions

In summary, the different straw return modes had varying impacts in the semiarid maize agroecosystems, and our research reflected the positive effect of straw returning on soil nutrient accumulation. Compared with CK, both the SR and ISR treatments increased the topsoil DOC content. Additionally, the ISR treatment increased the contents of TN, NO_3_^−^-N, NH_4_^+^-N, DON, and DOC in the subsurface soil. Compared with CK and SR, the ISR treatment also resulted in higher leaf and grain biomasses. GHG emissions varied under the different treatment conditions. The SR and ISR treatments led to increased soil CO_2_ emissions compared to CK, while both the SR and ISR treatments reduced the N_2_O emissions, with ISR being more effective in N_2_O emissions reduction. The correlation analyses indicated that the grain biomass was positively correlated with soil DOC. The soil CO_2_ emission was positively correlated with soil NO_3_^−^-N and TN. The soil N_2_O emission was positively correlated with soil DOC and negatively correlated with NO_3_^−^-N. ISR obtained a higher maize yield and alleviated the greenhouse effect by altering the soil DOC and NO_3_^−^-N contents. Therefore, the ISR technology holds significant application value and broad development potential for sequestering soil organic matter, enhancing soil fertility, promoting agroecosystem sustainability, and developing green agriculture. Such integrated management measures can improve soil quality and crop productivity while reducing environmental risks and providing effective technical support for sustainable agricultural development.

## Data Availability

Data will be made available on request.
